# Bound (“Glassy”) Rubber as a Free Radical Cross-linked Rubber Layer on a Carbon Black

**DOI:** 10.3390/ma11101992

**Published:** 2018-10-16

**Authors:** Alexey V. Kondyurin, Anastasia Yu. Eliseeva, Alexander L. Svistkov

**Affiliations:** 1 School of Physics, University of Sydney, 2006 Sydney, Australia; 2 ICMM UrB RAS, Academika Koroleva st, 1, 614013 Perm, Russia; anastasia_elis@mail.ru (A.Y.E.); svistkov@icmm.ru (A.L.S.)

**Keywords:** rubber, carbon black, glassy layer, graphite, free radicals, crosslinking, ellipsometry, FTIR

## Abstract

A model of rubber with a cross-linked rubber layer on a carbon black filler has been proposed. The cross-links are the result of free radical reactions generated by carbon atoms with unpaired electrons at the edge of graphitic sheets in a carbon black filler. The experimental study of the cross-linking reactions in polyisoprene was done on a flat carbonized surface after ion beam implantation. The cross-linking process in the polyisoprene macromolecules between two particles was simulated. The model with a cross-linked rubber layer on a carbon filler as a “glassy layer” explains the mechanical properties of the rubber materials.

## 1. Introduction

Rubber materials are widely used in modern industrial processes, devices, and machines: from tires for Formula 1 racing cars and thermoprotecting coating in solid rocket engines up to agriculture irrigation systems and elements of clothes [1,2,3,4,5,6]. However, the mechanical properties of the rubber materials are not well understood [7]. A rubber is a heterogenic material with high mechanical strength and deformability. Usually, the rubber contains an elastic matrix with embedded solid nanoparticles. The interaction between the elastic matrix and the particles play a key role in the mechanical properties of the rubber. From the beginning of the 20th century, the natural gum is filled with carbon black particles, which increased the tensile strength by 5–15 times and deformation when broken two to four times. Later, the same strength of the rubber with the carbon black was observed for artificial elastomers such as polybutadiene, polyisoprene, butyl rubber, styrene–butadiene rubber (SBR), ethylene–propylene rubber (EPR) and ethylene–propylene–diene rubber (EPDM).

The interface interaction between the rubber and the filler plays a significant role in the mechanical behavior of the rubber. A good illustration of the interface influence is a surface modification of silica particles in SBR rubber, which improves the mechanical properties of the rubber [8]. The strong influence of the interface interaction on the rubber mechanics was demonstrated on a sample of silica filler with a chemically modified surface [9]. A surface modification of the black carbon filler is not used, because the rubber with unmodified carbon black particles provides enough high strength and elasticity. However, the reason for such a good interface interaction is not clearly understood.

From early investigations, the sufficient interface interaction between the gum and black carbon particles is connected with the “bound rubber” effect. The formation of a specific “bound rubber” surrounding the carbon black particles has discussed since 1925 in a number of publications [7]. In the most recent publications, the “bounded rubber” is split to “tightly bounded rubber”, which is known in the literature as a “glassy rubber” and “loosely bounded rubber”. The “tightly bounded rubber” is characterized by its insolubility, high strength, and a very limited elasticity. The “loosely bounded rubber” can be dissolved under strong conditions (temperature/solvent/time), and remains elastic as a bulk rubber between the particles. In particular, the “loosely bounded rubber” forms the filaments between the particles. All together, the “bounded rubber” amount could achieve about 30% of whole gum in the filled rubber. The presence of the “glassy” rubber layer with low mobility over the particles and “loosely bounded rubber” over them is essential to explain the mechanical properties of the rubber [10,11].

The explanation of the rubber strength with the carbon black is based on a specific absorbance of the rubber macromolecules on the carbon black surface. A specific interaction between the macromolecules and graphitic rings is considered, and oxygen-containing groups are involved in the interaction. The “bound rubber” formation is connected with specific topological effects at the presence of the carbon black nanoparticles. However, all of the theories do not have solid experimental evidence, and cannot explain all of the mechanical properties of the rubber [7,10,12,13].

The chemically adsorbed rubber on the carbon black was observed before [12]; however, the chemical reaction between the rubber and the carbon black particles is considered as the free radical rubber macromolecules—which appeared due to chain scission during the mixing process—react with the hydrogen-terminated edges of the carbon black. Moreover, in some publications, the chemical reactions of the rubber with chemical groups on the carbon black surface is far less convincing, while only stable chemical groups such as carboxyl and hydroxyl groups, phenol, lactones, quinones, ketones, aldehydes, and hydroperoxides are considered on the carbon black surface. Other groups such as the free radicals were not considered [7].

We have to note that the investigation of the rubber macromolecule that is adsorbed on the carbon black nanoparticle is a very complicated experimental task for modern equipment, even today. The radius of the carbon black nanoparticle is comparable with the size of the macromolecule. The measurement of such a small and complicated object with high sensitivity has been an experimental problem up to now.

In the present study, we experimentally simulate the rubber “glassy” layer on a flat carbon surface. Such a model is a representative for black carbon filler in the rubber materials, and is a suitable experimental set for a detailed investigation of the adsorbed rubber layer on the carbon.

## 2. Hypothesis

**Figure 1:**
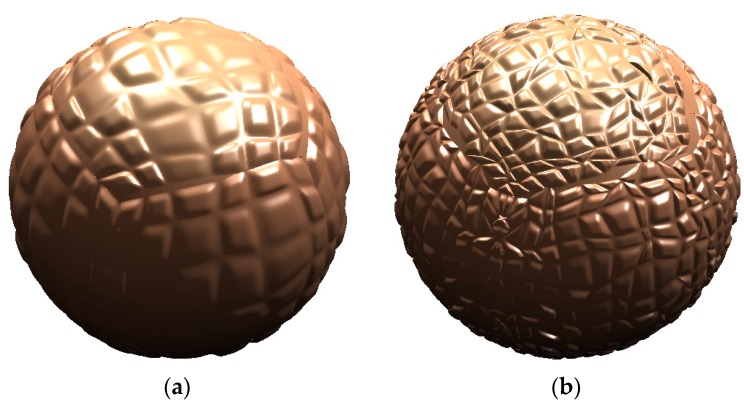
Computer geometrical model of carbon particle with low (**a**) and high (**b**) surface roughness.

**Figure 2:**
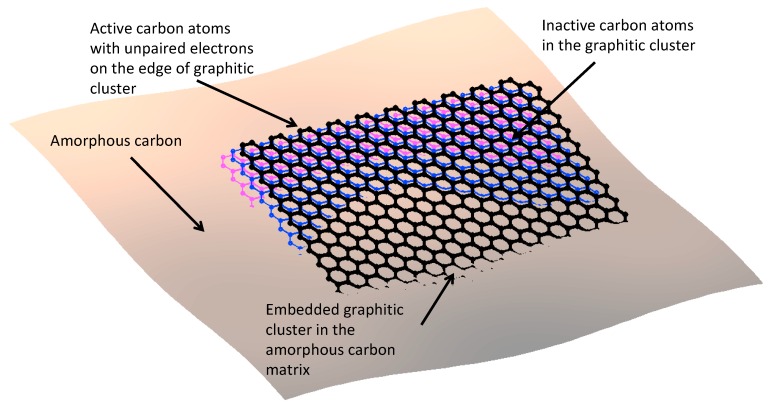
Illustration of graphitic cluster coming out the carbon particle on the surface. The active carbon atoms with unpaired electrons are on the edges of the graphitic sheet.

We consider the “glassy” layer of polyisoprene as a cross-linked layer on the carbon black particles. At first, carbon black particles should be considered in detail. The carbon black particles are not made out of a uniform material [14]. It consists of the amorphous carbon sp^3^ hybridization phase and the graphitic sheet sp^2^ hybridization phase, which is embedded into the amorphous phase, and is then cross-linked. These different phases make the surface of the particles very rough corresponding to the electron microscopy data [14]. For example, we consider two kinds of particles, with the smoother and with the rougher surfaces (Figure 1). The roughness of the surface is determined by the edges of the graphitic sheets, which are coming out of the surface (Figure 2). Therefore, the edges of the graphitic sheets are opened to the adsorbed polyisoprene macromolecules. The high roughness of the particle surface means a higher density of the edges of the graphitic sheets, and a higher free radical concentration on the surface.

The graphitic sheets are characterized with large π electron clouds. In general, the carbon atoms at the edges of the sheets have unpaired electrons, which cannot be shared with the neighboring carbon atoms. The presence of the unpaired electrons makes the edged carbon atoms actively join other atoms from the atmosphere, such as oxygen or nitrogen, or from any other molecules contacting the surface. However, the π electron cloud attracts the unpaired electron based on spin–spin interaction in such a way that the unpaired electron leaves the atom and gets shared with the π electron cloud for some time. When the unpaired electron leaves the edge atom to the cloud, the atom becomes chemically inactive. A larger cloud gives a longer time of sharing, and consequently a lower activity of the edge atom. The edge atom without the electron gets a positive charge, which attracts the electron back to the atom. The π electron cloud is deformed to partially compensate for the positive charge, but it cannot compensate for the whole charge. As the result, the dynamical equilibrium is achieved after some time once the unpaired electron is shared with the π electron cloud, while other times, the electron belongs to the atom. This equilibrium significantly decreases the activity of the edge carbon atom.

When the polyisoprene macromolecule is adsorbed on the carbon black surface, the macromolecule is in contact with the edge of the graphitic sheet for a long time. This is enough time to meet a condition when the unpaired electron comes back to the edge atom and brings back the high chemical activity of the edge.

**Figure 3:**
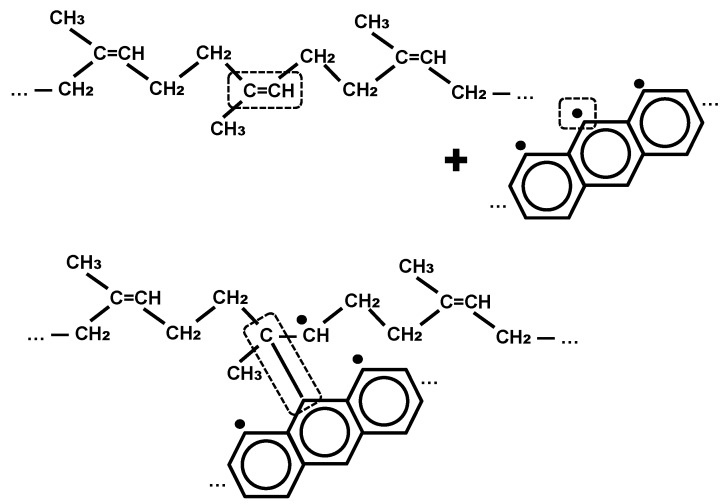
Chemical reaction of carbon atoms containing unpaired electrons on the edge of the graphitic cluster in the carbon black with a rubber macromolecule.

Such edges of the graphitic sheet react with the polyisoprene macromolecule, which is likely through opening the double carbon–carbon bond with the formation of a covalent bond between the polyisoprene macromolecule and the graphitic sheet. An example of this reaction is presented in Figure 3.

**Figure 4:**
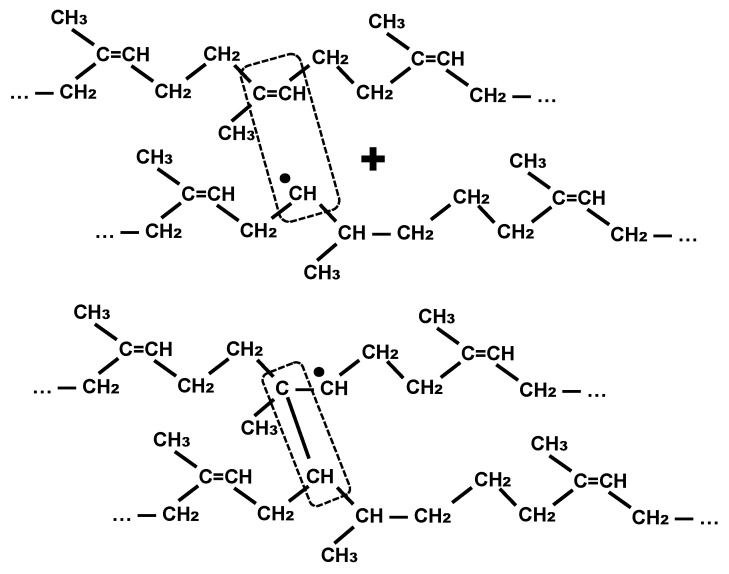
Chain reaction of cross-linking process in the polyisoprene macromolecules caused by free radicals migrated from the carbon black particle.

It’s less likely that the hydrogen can be subtracted from the macromolecule and the free radical that’s appeared at the macromolecule can join another carbon atom at the edge of the graphitic sheet or migrate further along the polyisoprene macromolecule. The migrated free radical could cause a formation of cross-links between the polyisoprene macromolecules from a distance from the surface of the black carbon particles. An example of this reaction is presented in Figure 4.

**Figure 5:**
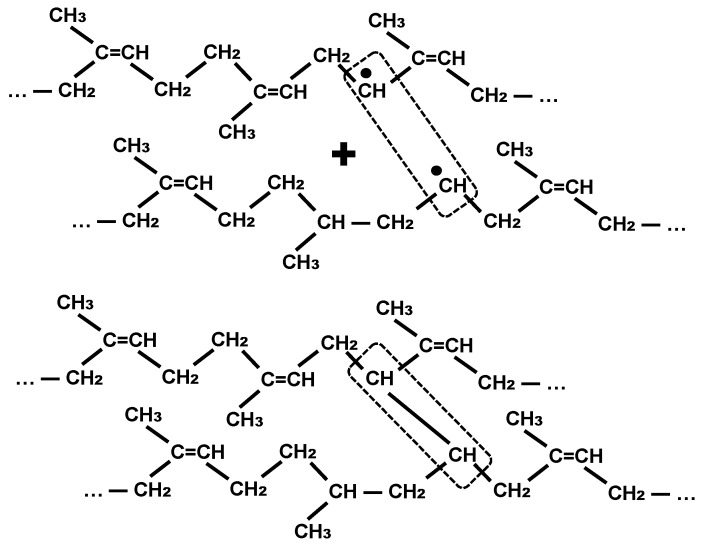
Quenching of the free radicals in polyisoprene macromolecules.

The migrated free radicals can meet and collapse with the formation of the cross-link between the chains of the polyisoprene macromolecule (Figure 5). All of these chemical processes have a probability, and are too complicated to simulate in detail. We have to underline here that the free radical migration is based on swopping hydrogen atoms from a neighboring group, but not any carbon atoms moving or any electron transfers.

To prove our hypothesis, the investigation of such chemical reactions on the carbon black particles is too complicated, and cannot give us the desired amount of experimental accuracy. We have simulated the carbon black particle surface with a carbonized flat surface on silicon wafer, and investigated the polyisoprene layer on that surface. 

## 3. Materials and Methods

These experiments are aimed to simulate the polyisoprene (PI) behavior on black carbon filler in rubber materials. The carbon flat surface was obtained from polystyrene (PS) coating carbonized with a high-energy ion beam. The polystyrene coatings of 120-nm nominal thickness were prepared by spin-coating at 2000 rpm using a SCS G3P-8 Spincoater onto (100) silicon substrates (20 × 20 mm, 0.610–0.640 mm thickness, P-doped to 10^4^–2 × 10^4^ ohm·cm, and polished on one side, Topsil, Sherman, TX, USA). The spin-coating solution consisted of polystyrene (Austrex 400 from Polystyrene Australia Pty. Ltd., Sydney, NSW, Australia) dissolved to a concentration of 20 g/L in toluene (CAS 108-88-3, Sigma-Aldrich, Castle Hill, Australia). A uniformity of the PS coating was observed due to the blue color distribution over the sample area. The samples with uniform coating were used for the following experiments. After spinning, the coating was treated by PIII for 800 s to get a completely carbonized coating. The nominal thickness of the carbonized coating was 100 nm.

The radio-frequency (13.56 MHz) plasma was generated to get the ions for plasma immersion ion implantation (PIII). An ENI ACG generator (ENI, MKS, Andover, MA, USA) with a matching box Comdel CPM-1000 generated the plasma power. The nitrogen gas plasma power of 100 W power with a reverse power of 12 W when matched was applied. The ultimate pressure of the vacuum chamber measured with a Pfeifer vacuum gauge was 10^−5^ Torr provided by XDS-10 scroll pump (Edwards, Burgess Hill, UK) with a nEXT 400D turbo molecular pump. The working pressure of nitrogen gas during the implantation process was 2 × 10^−3^ Torr, which was provided by an flow controller (MKS, Andover, MA, USA). The ions were extracted from the plasma and accelerated to the polystyrene target by the application of 20-kV bias pulses of 20 μs duration to the sample holder at a frequency of 50 Hz. The high voltage pulses were provided by a PI^3^ generator designed by the Australian Nuclear Science and Technology Organization (ANSTO, Sydney, NSW, Australia). The silicon substrates were mounted on a metal substrate holder that was 150 mm in diameter. Ion implantation occurred through a metal grid that was mounted over the 50 mm samples and electrically connected to the holder. The uniform fluence area was a circle that was about 100 mm in diameter.

The measurement of plasma parameters was done with using a Langmuir probe. The Langmuir probe was positioned over the substrate electrode and consisted of a 0.20-mm diameter tungsten wire passing through a sintered alumina ceramic tube inserted into a stainless steel tube grounded to the chamber. An electronic block and software from Hiden Analytical Ltd. (Warrington, UK) was used for the measurement and analysis of the data. The floating potential of the substrate electrode was 40–45 V during the plasma treatment. The plasma density was 2.6 × 10^9^ ions/cm^3^.

Ion fluence estimates were obtained from the number of high voltage pulses multiplied by the fluence corresponding to one pulse. The procedure of fluence estimation is described by Kondyurin in [15]. Following the previous results obtained using polyethylene [16] and using the method of fluence estimation described by Kondyurin in [15], one second of PIII treatment in the system configuration that was used here gives a fluence of 1.25 × 10^13^ ions/cm^2^. The samples were treated for durations of 800 s, corresponding to implantation ion fluences of 10^16^ ions/cm^2^.

The samples were stored in hermetic LDPE boxes protected from dust and light in an air-conditioned laboratory (23 °C). The surface of the carbonized layer was not in contact with any materials.

Some carbonized samples were soaked in 10% TEMPO (2,2,6,6-Tetramethyl-1-piperidinyloxy, free radical, CAS 2564-83-2, Sigma-Aldrich, Castle Hill, Australia) solution in ethanol (Sigma-Aldrich, Castle Hill, Australia). After soaking, the samples were washed three times in ethanol for 1 min each time in a new portion of 10 mL of ethanol, and were dried overnight before the measurements. 

The cis-polyisoprene purchased from Sigma Aldrich (CAS 104389-31-3, average Mw ~ 38,000 by gel-penetrating chromatography) was dissolved to a concentration of 10 g/L in toluene. The polyisoprene was spun on the top of the carbonized coating under the conditions that were described above. The nominal thickness of the polyisoprene coating was 100 nm. A uniformity of the polyisoprene coating was observed because of a yellow–green color distribution all over the sample area. The samples with uniform coating were used for the following experiments.

Some samples were annealed in a vacuum oven Binder VD53 (Binder GmbH, Tuttlingen, Germany). The temperature accuracy was ±1 °C. The vacuum was provided by a rotary vacuum pump.

The samples were washed in toluene and heptane (Sigma-Aldrich, Castle Hill, Australia) in an ultrasound bath (Power Sonic 405, Thermolone Scientific, Wetherill Park, Australia) for 5 min. Then, the samples were washed in a new portion of the same solvent, and dried before the investigation.

The Raman spectra of samples were recorded on the triple monochromators spectrometer Jobin Yvon with a CCD matrix as a detector cooled by liquid nitrogen. The spectra were excited by YAG laser with a second harmonic of a 532.14-nm line. The diffraction grid was 1800, the slit was 200 nm, the objective was 100, and the integration time of one scan was 1 s. The number of scans was varied from 50 to 300 in order to obtain a good signal/noise ratio. Irradiation of the sample and the collection of scattered light was done using a microscope to get micro-Raman spectra from the surface layer.

Electron paramagnetic resonance (ESR) spectra were recorded on a Elexsys E500 ESR spectrometer (Bruker, Billerica, MA, USA) at room temperature. The microwave frequency of 9.75 GHz and a central magnetic field of 3480 G were used. The spectrometer was calibrated using DPPH (α,α_0_- diphenyl-b-picrylhydrazyl) standard. The polystyrene film was rolled and placed in a quartz glass capillary. The capillary was placed into a resonator of the ESR spectrometer. The spectra of the untreated polystyrene film and the used quartz tube were recorded as a control. A number of spectra scans and magnetic field steps were selected to get a low noise signal of the unpaired electrons with sufficient spectral resolution.

FTIR transmission spectra were registered on Digilab FTS7000 (Agilent, Melbourne, Australia) and Bomem spectrometers (Bomem ABB, Quebec City, QC, Canada).The spectra of an initial silicon wafer and carbonized PS layer were used for subtraction from the spectra of the samples. The number of the scans was 1000, and resolution was 4 cm^−1^. GRAMS software was used for the spectra analysis. The optical density of the spectral lines that were associated with particular bond vibrations were used to quantify the structural changes following the Bouger–Lambert law.

The thicknesses and optical constants of the spun polymer films were determined using a Woollam M2000V spectroscopic ellipsometer. Ellipsometric data was collected for five angles of incidence: 55°, 60°, 65°, 70°, and 75°. The experimental data were fitted with a model consisting of a Cauchy layer with absorbance on top of the silicon substrate with an SiO_2_ layer. The fitted model of the carbonized PS layer was used for following fitting of the polyisoprene sample data.

## 4. Results

The PIII treatment carbonizes the polystyrene thin layer on the silicon wafer. However, the industrial carbon black filler is made up of a different precursor, and had a different process. Despite having common carbon properties, we characterized the carbon coating after PIII, and compared it with the known black carbon properties from the literature.

### 4.1. Characterization of the Carbonized Layer

The structure of the carbon black particles depends on the method of preparation. A general process of the carbon black is a carbonization of the carbon-containing precursor at a high temperature. In general, the carbon black consists of carbon (95%–99%) and other elements such as oxygen, nitrogen, sulfur, and hydrogen in different concentrations [17]. The carbon black that was used in rubbers contains a mixture of carbon phases of sp^2^ and sp^3^ hybridizations with different sizes (from 1 nm to 100 nm) of the graphitic clusters. The graphitic plains in carbon black are disoriented and linked with the amorphous carbon structures between them. The carbon black particles usually have a size of 10–500 nm with a high effective surface area (25–150 m^2^/g) [18]. It indicates that the particles have non-regular shape and a pore structure with lower density than the pure graphite or diamond.

**Figure 6:**
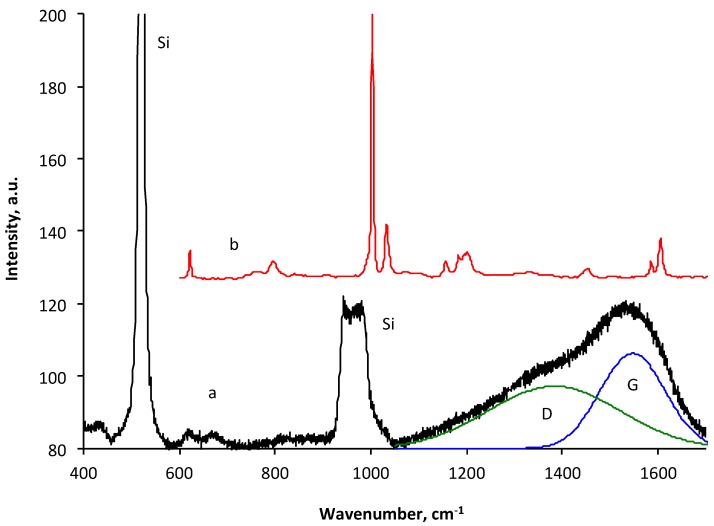
Raman spectra of plasma immersion ion implantation (PIII) treated polystyrene on silicon wafer (a) and untreated bulk polystyrene (b). PIII treatment was done with 10^16^ ions/cm^2^ fluence of 20 keV energy N^+^ ions.

The carbon coating generated by the PIII of the polystyrene layer in our experiments is characterized with a smooth surface of about 1 nm or less RMS (root mean square). The Raman spectrum (Figure 6) of the carbonized coating contains two peaks, which are associated with the carbon structure D-peak and G-peak. The peaks of polystyrene are not observed in the spectra of the carbonized coating. The silicon peak is due to the underneath of the silicon wafer substrate. The fitting of the Raman band with two Gauss functions gives the D-peak position at 1383 cm^−1^ and the G-peak position at 1547 cm^−1^. The ratio of the integral D-peak to the integral intensity of the G-peak is 1.32. These parameters correspond to the nanocrystalline graphite structure with a characteristic graphitic cluster size of 3.3 nm [18]. For a comparison, the Raman spectra of the carbon black N330 with a surface area of around 78 m^2^/g obtained from Cabot shows the lines D-peak position at 1355 cm^−1^ and G-peak position at 1586 cm^−1^ [19] with a 514.5-nm laser line. Other industrial black carbons give the following positions for D and G peaks correspondingly: 1364 and 1539 for Printex XE2 (Degussa), 1371 and 1559 for Printex L (Degussa), 1359 and 1548 for Merck (Merck), and 1371 and 1551 for Vulcan XC-72 (Cabot) [20]. These parameters correspond to the characteristic graphitic cluster size between 1.4–5.6 nm. Therefore, the carbonized coating generated by the PIII of the polystyrene layer in our experiments has similar graphitic clusters as in the industrial carbon black.

**Figure 7:**
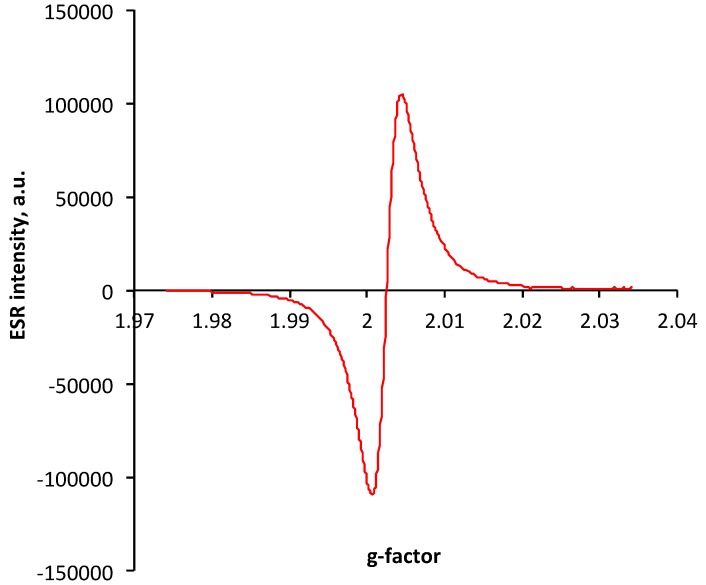
ESR spectra of polystyrene film treated by 20 keV nitrogen ions with a fluence of 10^16^ ions/cm^2^.

The presence of unpaired electrons in PIII-treated polystyrene was proven with an ESR spectra. The strong clear peak with a center at a 2.0025 g factor is observed in polystyrene film treated as a satellite sample to the polystyrene coating on the silicon wafer (Figure 7). The position of the peak is close to a free electron g factor (2.0023) [21]. It corresponds to the unpaired electrons delocalized on π electrons of the condensed aromatic structures. The same position and shape of the peak was observed in the ESR spectra of carbon black [22,23,24,25].

**Figure 8:**
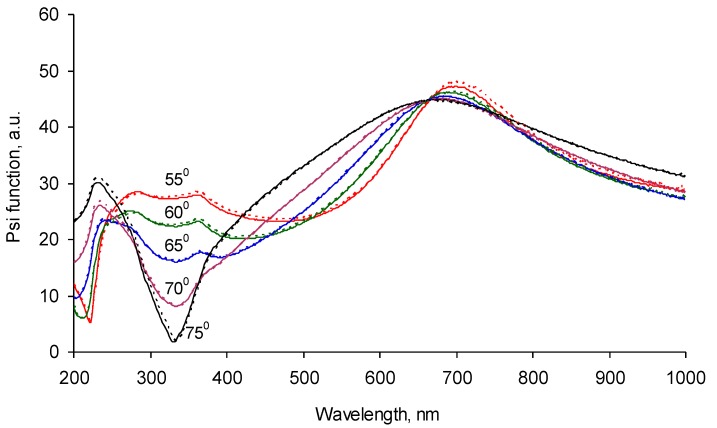
Ellipsometry Ψ function of the silicon wafer sample with the carbonized coating on the top recorded at 55, 60, 65, 70, and 75 degrees of incident angles. The continuous lines are experimental spectra. Dotted lines are the fitting model.

**Figure 9:**
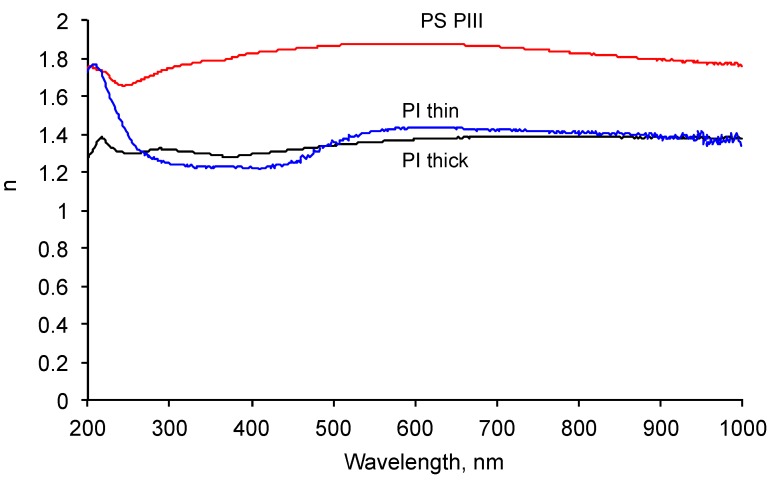
Ellipsometry data fitting result of the refractive index: red line is for carbonized polystyrene (PS) PIII coating on silicon wafer as the analog of carbon black; black line is for the spun thick (100-nm) polyisoprene layer on the top of the carbonized coating; blue line is for the thin (10-nm) polyisoprene layer on the carbonized coating after washing in toluene and hexane.

The optical properties were measured with ellipsometry in the wavelength range of the UV and visual waves. The ellipsometry Ψ function for five angles of reflection is presented on Figure 8. The fitting of the experimental data was done with a Cauchy model with absorbance for the carbonized layer on top of the SiO_2_ sub-layer and the Si bulk layer of the silicon wafer. The functions n and k for the carbonized layer were found to be unique for these experimental datasets. The refractive index of the carbonized layer was found to be in the 1.7–1.9 range in the 200–1000 nm spectral diapason with a weak dispersion in the 200–300 nm spectral diapason (Figure 9).

The observed refractive index is significantly higher than the refractive index of the polystyrene (1.4–1.5). This refractive index (1.9) at a wavelength of 550 nm is in the range for the refractive index of carbonized structures such as 2.1 for DLC film [26], 2.1–2.2 for graphite, 2.35 for diamond, 1.78–1.98 for coal, and about 2 for carbon black [27].

The prepared carbonized layer was tested and found to be insoluble in ethanol, toluene, heptane, acetone, and water. The layer is stable against scratching in experiment manipulations and stable when heated up to 150 °C. 

The resulted carbonized layer has some similarities with the carbon black, including: graphitic and amorphous fractions of the carbon, unpaired electrons stabilized on p electrons of aromatic condensed structures, and optical properties. However, the curvature of the surface is different. With such assumptions, the carbonized layer was accepted as a flattened analog of the carbon black particles in the following experiments with the rubber.

### 4.2. Polyisoprene Layer on the Carbonized Layer

**Figure 10:**
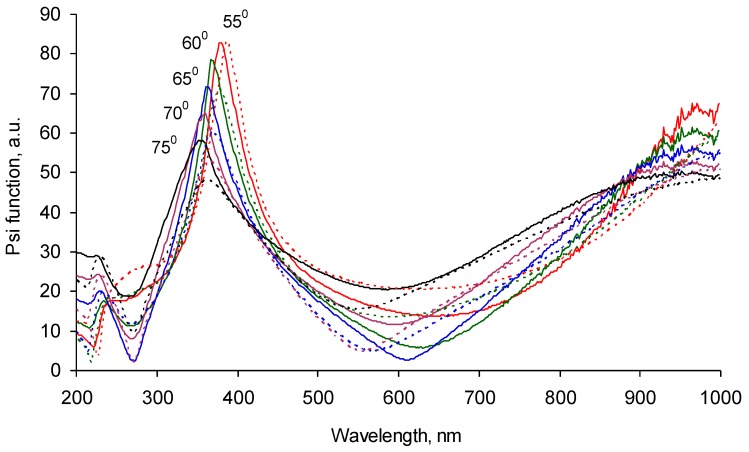
Ellipsometry Ψ function of the silicon wafer sample with the carbonized coating and polyisoprene spun (thick) layer on the top recorded at 55, 60, 65, 70, and 75 degrees of incident angles. The continuous lines are experimental spectra. Dotted lines are the fitting model.

The polyisoprene-spun layer was smoothly distributed over the carbonized coating. The ellipsometry spectrum shows a wavelength shift of the Ψ function peak from 700 nm to 350–400 nm (Figure 10). The experimental ellipsometry spectra were fitted with a multilayer model of Si as bulk, SiO_2_, and carbonized layers with fixed optical properties and thicknesses calculated from previous measurements without any polyisoprene layers or any polyisoprene layers on the top. The polyisoprene layer was fitted by a Cauchy model with absorbance. The calculated refractive index of the polyisoprene was in the range of 1.3–1.4 (Figure 9). The calculated thickness of the polyisoprene layer was 120 nm. Such a sequential measurement of silicon wafer, silicon wafer with carbonized layer, and silicon wafer with carbonized and spun polyisoprene layer was done for each sample. The correlation coefficient matrix was checked at the fitting to exclude the cross-influence of the fitted parameters. Such a way gives stable, fitting results for all of the polyisoprene samples.

**Figure 11:**
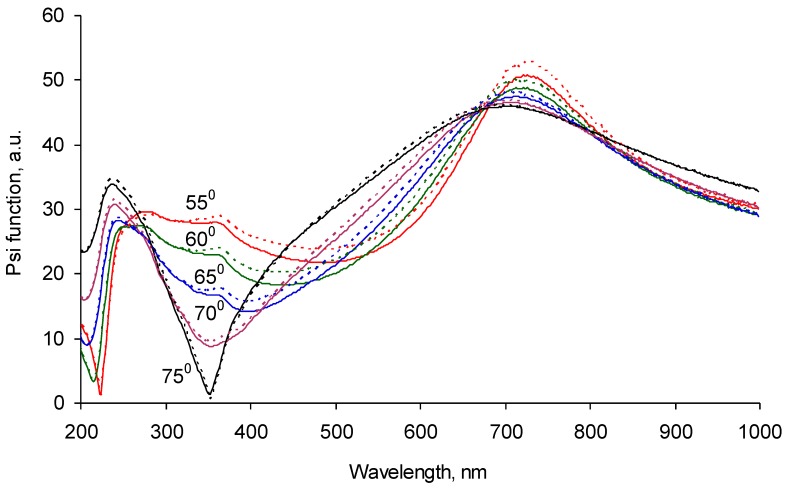
Ellipsometry Ψ function of the silicon wafer sample with the carbonized coating and polyisoprene spun and then washed with (thin) layer on the top recorded at 55, 60, 65, 70, and 75 degrees of incident angles. The continuous lines are experimental spectra. Dotted lines are the fitting model.

The ellipsometry spectrum of the same sample-washed toluene and heptane shows a backward shift of the Ψ function peak from 350–400 nm to about 700 nm of wavelength (Figure 11). The fitting of the experimental data with a multilayer structure consisted of a silicon wafer, silicon oxide, carbonized layer, and polyisoprene layer simulated with a Cauchy model with absorbance gave a thickness of the remained polyisoprene layer in a range of 6–14 nm for different samples. The refractive index of the remaining polyisoprene layer (Figure 9, blue curve) was similar to the spun thick polyisoprene layer (Figure 9, black curve) in the experiment presented in Figure 10 with a slight difference of a shorter wavelength region of 200–250 nm. The remained polyisoprene layer shows dispersion in this region of the refractive index. When the experimental data was fitted with the optical constants of the polyisoprene layer calculated from the spectra presented above, the result of the top layer thickness was similar. The repeated washing of the samples in toluene and heptane did not change the ellipsometry data and the fitting results.

The stability of the fitting results was proven with multiple unsuccessful attempts to fit the experimental data without the top polyisoprene layer. Also, an attempt to fit the experimental data with only one layer of carbonized coating on the silicon wafer was not successful.

**Figure 12:**
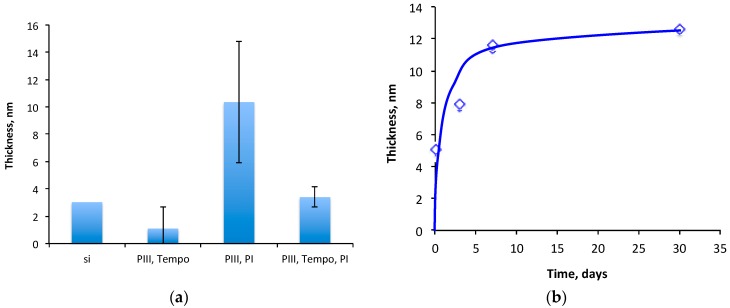
Thickness of the top remained layer over the carbon surface: (**a**) Thickness of the top remained layer over the carbon surface: “si” is a silicon wafer that was only washed with heptane; “PIII, TEMPO” is the silicon wafer with carbon coating treated by TEMPO and washed with heptane; “PIII, PI” is the silicon wafer with carbon coated by polyisoprene, kept 1 h at 23 °C, and washed with heptane; “PIII, TEMPO, PI” is the silicon wafer with carbon treated by TEMPO, then coated by polyisoprene, kept 1 h at 23 °C and washed with heptane; (**b**) Thickness of remained polyisoprene layer on the carbon coating in dependence on the storage time of the spun thick polyisoprene on the carbonized coating before the washing.

The thickness of the polyisoprene layer that remained on the carbonized layer was compared with the top layer thickness in the control samples (Figure 12a). The thickness of the remained polyisoprene layer on silicon wafer without the carbonized coating is about 3 nm. Such a thickness corresponds to a residual layer of hydrocarbon contamination, which can remain on a silicon wafer after washing in organic solvents (1–2 nm). The same thickness on top layer is observed after the attachment of TEMPO on the carbonized coating. 

The pre-attachment of TEMPO to a carbonized coating significantly decreased the thickness of the remaining polyisoprene layer. When the carbonized coating was pre-treated by TEMPO, and then the polyisoprene was spun and washed out from the carbonized coating as in the experiment described above, the thickness of the remained polyisoprene layer was 2 nm.

**Figure 13:**
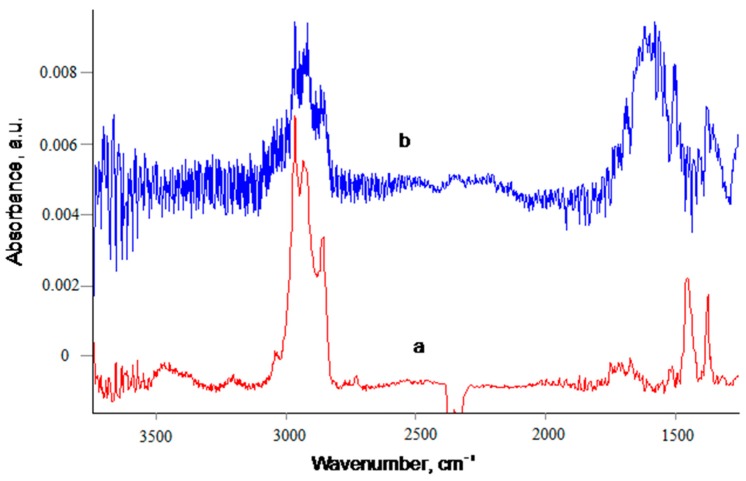
Fourier transform infrared (FTIR) transmittance spectra of silicon wafer with 100-nm spun PI (a) and silicon wafer with carbonized coating with a spun 100-nm polyisoprene layer kept for 30 days at 23 °C and washed with toluene (b). The spectra of silicon wafer and PS carbonized layer are subtracted. The spectra are offset for a good view. The absorbance scale for (b) spectrum is zoomed in 60 times.

The chemical composition of the remained polyisoprene layer was proven with transmission Fourier transform infrared (FTIR) spectra (Figure 13). The spectrum of the spun polyisoprene layer with a thickness of 100 nm on the silicon wafer is presented on Figure 13a. The spectrum shows 3030 cm^−1^, 2963 cm^−1^, 2925 cm^−1^, and 2855 cm^−1^ lines interpreted as stretch C–H vibrations in =CH-, -CH_2_- and -CH_3_ groups, the 1670 cm^−1^ line was interpreted as stretch C=C vibrations and 1453 cm^−1^ and 1376 cm^−1^ lines were interpreted as bending C–H vibrations in the polyisoprene macromolecule. Besides that, the weak broad lines with a center position near 3450 cm^−1^ and 1720 cm^−1^ are interpreted as a result of oxidized polyisoprene macromolecules under environmental conditions. The spectrum of the remained polyisoprene layers after washing shows similar lines of the polyisoprene macromolecule with a much lower intensity, corresponding to lower thicknesses of the layer. Additionally to the lines related to polyisoprene, the strong broad line with a center of 1610 cm^−1^ was observed and interpreted as C=C vibrations.

The thickness of the remained polyisoprene layer on the carbonized coating increases with the time of contact (Figure 12b). The thick polyisoprene layer (100-nm) was kept on the carbonized coating from one hour to one month at 23 °C, and then washed out with toluene and heptane. The thickness of the remained polyisoprene layer increases asymptotically from 5 nm for 1 h to 12 nm for the month storage time. The saturation level was achieved after a week of the storage time.

**Figure 14:**
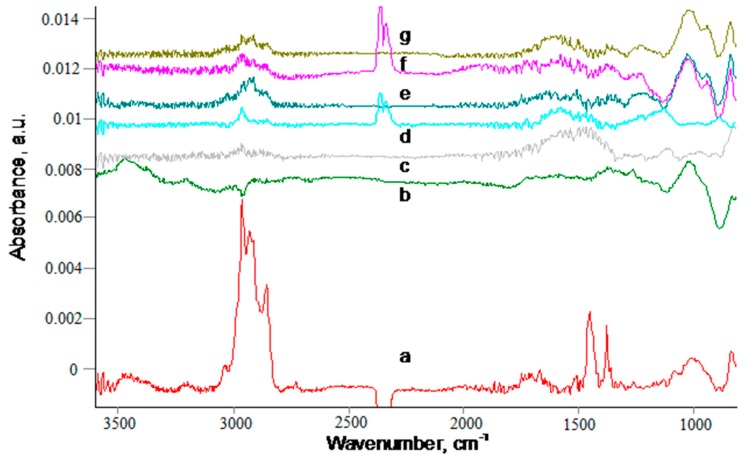
FTIR transmittance spectra of silicon wafer with spun 100-nm PS, PIII N+ 20 keV 800 s, spun 100-nm polyisoprene, kept at 23 °C, and washed with toluene/heptane. (a) spun 100-nm PI layer on Si, (b) spun PI on Si and washed with toluene, (c-f) PI on carbonized PS kept at 23 °C (c) 1 h, (d) 90 min, (e) three days, (f) seven days, (g) 30 days and then washed with toluene. The spectra of silicon wafer and PS carbonized layer are subtracted. The spectra are offset for a good view.

The composition of the polyisoprene layer stored on the carbonized coating did not change with the storage time (Figure 14). The lines of the polyisoprene macromolecules were observed in all of the samples with the polyisoprene layer, while for the shorter storage time, the intensity of some lines in the middle infrared region were at the level of noise. For such samples, the presence of polyisoprene can be only observed a short wavelength region (Figure 14c,d). For a comparison, the spectra of silicon wafer without carbonized coating (Figure 14b) did not show the lines related to the remained polyisoprene layer after washing.

### 4.3. Annealing Polyisoprene Layer

**Figure 15:**
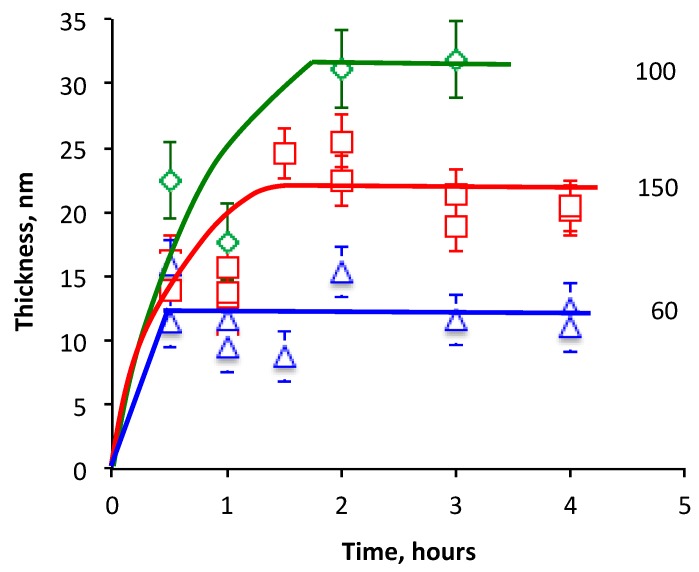
Thickness of remained polyisoprene layer on the carbon coating in dependence on the storage time of spun polyisoprene on the carbonized coating at different storage temperatures (°C).

The vulcanization process in the rubbers usually occurs at elevated temperatures. The interaction of the polyisoprene and the carbonized coating under the vulcanization conditions were simulated with the annealing of the spun thick layer of polyisoprene on the carbonized coating. An oxidation of the polyisoprene layer during annealing was prevented by vacuum conditions. The thickness of the remained polyisoprene layer on the carbonized coating in dependence storage time and temperature is presented in Figure 15.

The thickness of the remaining polyisoprene layer grew up with the annealing time as it was observed at 23 °C. However, the saturation level was achieved faster and higher at elevated temperature. The thickest polyisoprene layer of about 32 nm is observed at a 100 °C annealing temperature. At 150 °C, the saturation level became lower. Two hours are enough to get the saturation level at all of the temperatures.

**Figure 16:**
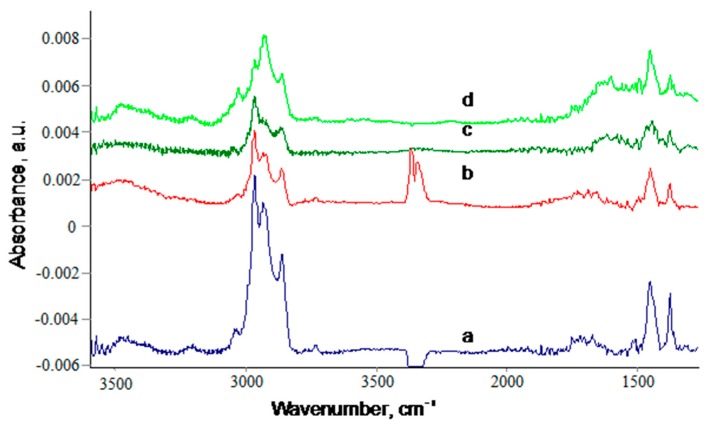
FTIR transmittance spectra of silicon wafer with carbonized coating and spun 100-nm Polyisoprene (a), annealed at 150 °C and wash with toluene and heptane (b–d). Time of annealing was 1.5 h (b) 3 h (c), and 4 h (d). The spectra of silicon wafer and PS carbonized layer are subtracted. The spectra are offset for a good view.

The composition of the remained polyisoprene layer after annealing is similar, and does not change with a longer annealing time and higher temperature (Figure 16). The same spectral lines of polyisoprene macromolecule vibrations and additional C=C stretch vibrations are observed in the spectra.

The experiments with the spun polyisoprene showed that the carbonized coating provides a stable layer of polyisoprene on the surface. The layer could not be dissolved with the solvents that were suitable to dissolve the bulk polyisoprene. The same effect was observed after chemical cross-linking of the polyisoprene after vulcanization of the rubber under sulfur-containing vulcanizing agents and peroxide agents. In both cases, the vulcanization occurred on free radical reactions of the opening double bond in residual isoprene unit.

The vulcanization process is a chemical reaction with a rate depending on the temperature. The same thickness grown is observed in terms of the time and temperature of the annealing. At the same time, the FTIR spectra showed that the chemical composition of the layer corresponded to polyisoprene macromolecules, as it was observed for the vulcanized rubber. The similarity of the polyisoprene behavior on the carbonized coating and in the rubber with carbon filler could be interpreted as the similar chemical process of the cross-linking in the polyisoprene.

The formation of the glassy layer of polyisoprene was observed in our previous experiments on stiffness measurements of the polyisoprene-spun layer on the carbonized coating. Atomic force microscopy (AFM) measurements showed that the polyisoprene layer of 45 nm and thicker are soft, while the layers of 18 nm, 9 nm, and 5 nm are gradually stiffer with the decreasing thickness [28], while in that publication, we did not consider the free radical mechanism of the polyisoprene stiffening.

Similar cross-linking was observed in acrylamide gel coating on the plasma and the ion beam activated surface of the carbon-containing substrates. The same free radical mechanism is responsible for the protein attachment on the PIII-treated polymer surface and plasma polymerized coatings. The same free radical mechanism is responsible for the cross-linking in the interface of the adhesion joints in the rubber and the other polymer materials. Therefore, these experiments and the known literature data show that the polyisoprene can form the cross-linked layer on the carbon black particles in the rubber materials due to the free radical reactions on the graphitic on the sheet edges.

### 4.4. Modeling the Interactions between Two Carbon Black Particles

**Figure 17:**
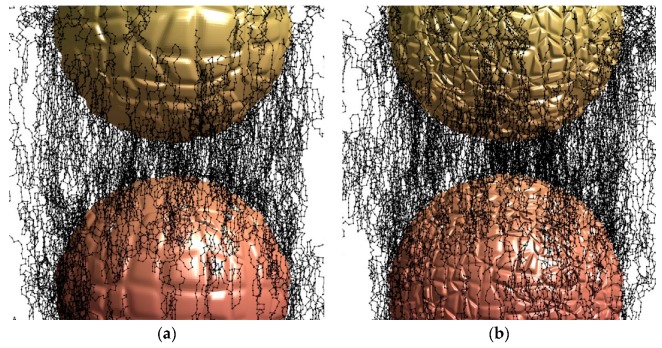
Computer model of the cross-links in polyisoprene between the carbon black particles with low (**a**) and high (**b**) surface roughness.

We consider an example of this chemical reaction for an analysis of the cross-links in the polyisoprene between two carbon black particles. The diameter of particles is assumed to be 30 nm. The distance between the particles is assumed to be 5 nm. The space between the particles is filled with polyisoprene macromolecules. The cross-links between the macromolecules are calculated with a random motion algorithm. The starting point of the cross-links was always from the surface of the particles. It was assumed that 60% of the free radicals on the carbon particle surface are quenched due to reactions with atmospheric oxygen or other contaminations from the environment. The minimal distance between the cross-links is assumed to be 0.65 nm, which corresponds to a monomer unit of the polyisoprene macromolecule. The step of the random motion was also accepted as 0.65 nm. The two free radicals could be quenched when they met. Also, the free radical could be quenched when it meets another active additive or dissolved oxygen, and reacts without the cross-link with a probability 3%. The calculations were stopped when all of the radicals on the surface were completely spent. The results of the calculations for the high and low roughness of the carbon black particles are illustrated on Figure 17.

**Figure 18:**
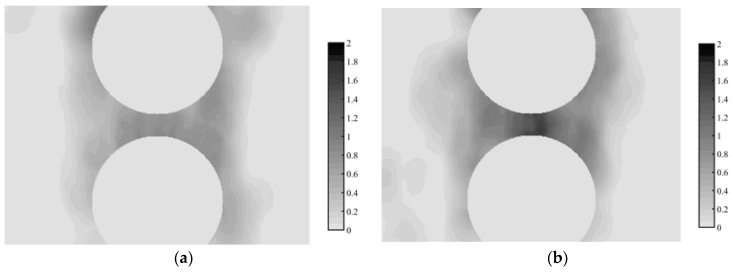
Cross-link density in polyisoprene between carbon black particles with low (**a**) and high (**b**) surface roughness. The darker color corresponds to higher density of the cross-links. The scale of darkness in dependence on the cross-link density in crosslink per 1-nm^3^ units is presented on the right side of the image.

The following calculations of the mechanical properties were done based on a continual model of the rubber. The distributions of the individual cross-links were recalculated to a distribution of the cross-links’ density in the polyisoprene rubber. A volume for averages of the cross-links was 5 nm. The sections of the cross-link density distribution for the high and low roughness of the carbon black particles are presented on Figure 18. The sections are presented on a one plane through the centers of the particles, while the results are relatively symmetrical to the axis through the centers of the particles, while some of the cross-links are observed at a far distance from the particles.

**Figure 19:**
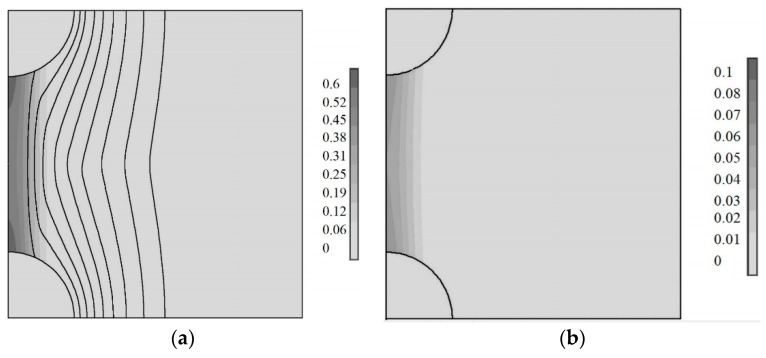
The Young’s modulus equal lines (MPa) and the stress distribution (dark intensity, MPa) between two carbon black particles with cross-links due to the free radical reactions (**a**) and without cross-links due to the free radical reactions (**b**) at the mechanical loading in the rubber.

In the simplest assumption, the cross-links change the Young’s modulus of the polyisoprene between the particles significantly. The Treloar [29] potential was used to calculate the contribution of the added cross-links to the modulus of the rubber:

E = 6 × (N_1_ + N_2_) × kT (1)

where N_1_ is the cross-link density in the polyisoprene rubber without the carbon black particles, N_2_ is the density of cross-links due to the presence of the carbon black particles, k is the Boltzmann constant, and T is the temperature. The formula corresponds to a case when the number of polyisoprene units is higher than the cross-links by a factor of at least two; therefore, at least four polyisoprene units must surround one cross-link as a minimum. The temperature assumed is 293 K. The modulus of the rubber without carbon black particles is assumed to be 1 MPa. Following the calculations above, the maximal crosslink density is 1.2 cross-links per nm^3^ for low roughness particles and 1.7 cross-links per nm^3^ for high roughness particles. Following these assumptions, the modulus between the low roughness particles is 9.7 MPa. Equal lines of the Young’s modulus are shown on Figure 19. For a comparison, the case of a uniform distribution of the cross-links due to vulcanizing of the rubber is also presented (b).

The strain–stress calculations were based on a cell consisting of these carbon black particles with polyisoprene rubber in between them. The cells are regularly distributed in the material, which expands along the line through the centers of the particles. The initial gap between the particles is 5 nm. The tangent stress is assumed as zero. The results of the calculations are presented in Figure 19. The images correspond to an expanded cell, which is 5 nm.

Two cases were considered: with additional cross-links due to free radical reactions (a) and without additional cross-links (b). Just in case, the cross-link density is assumed to be uniformly distributed over the whole rubber due only to vulcanizing reactions. The stresses are concentrated in between the particles. However, the dark contrast corresponding to the stress intensity is much higher in this case with additional cross-links (0.6 MPa) than without them (0.1 MPa). Therefore, a force between two particles is much higher, where in this case the additional cross-links are then without them.

**Figure 20:**
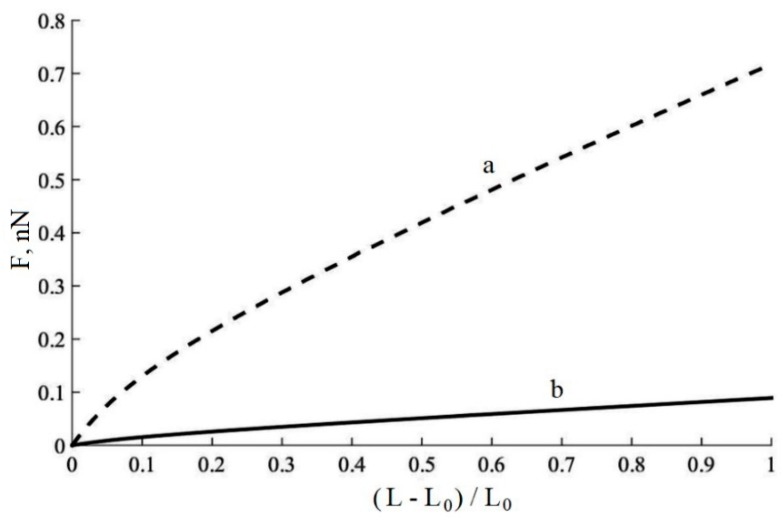
Force between two carbon black particles in the rubber in dependence on relative distance (L) between the particles with cross-linking in the polyisoprene macromolecules due to free radical reactions (a) and without cross-links (b).

The force curve in dependence on the distance between the particles is shown in Figure 20. The force between the particles is about 10 times higher with the presence of additional cross-links between the particles than without the additional cross-links.

## 5. Discussion

The experiment has shown that the carbon black particles can initiate the cross-linking reactions in the adsorbed polyisoprene layers. The nature of these reactions is a pool of free radicals, which come from the edges of the grapheme planes where the edge carbon atoms have unpaired electrons. When the polyisoprene macromolecule is adsorbed on the carbon black surface, the carbon atom of the grapheme edge can substitute for the hydrogen or disconnect the double bond in the polyisoprene macromolecule. As the result, the free radicals can migrate to the macromolecule, and cause the chains of the free radical reactions. Such free radical reactions are responsible for a formation of gel structures in the rubber in long time experiments on a mixture of the natural polyisoprene with carbon black particles. Here, we have to highlight that the considered free radical reactions occur in the rubber before the vulcanization process, where the free radical reactions play the key role in the cross-linking of the bulk rubber. However, the vulcanization processes at a high temperature can accelerate the free radical reaction near the carbon black surface, as it is observed in the annealing experiments.

The chemical reactions with the free radicals in polymers are complicated and have random character. The results of the reactions can be cross-linking of the different chains in the macromolecule or cross-linking between macromolecules, double-bond formation, oxidation at the presence of atmospheric oxygen, scission of the macromolecules on short chains, or up to monomers. In particular, such oxidation of the adsorbed polyisoprene layer of the carbon surface is well observed in our experiments. Such a significant transformation of the chemical structure can change the interactions between the polymer macromolecules and between the macromolecules and the carbon black surface. The increasing the interactions between the new polar groups of the polyisoprene macromolecules and the polar groups of the carbon black particles can be expected, as well as a decrease in the interactions between the oxidized and virgin polyisoprene macromolecules with the result of more easily sliding them to each other at loading. 

When the cross-linking mechanism dominates for particular polymer, in particular, the polyisoprene is predominantly a cross-linkable polymer, the mobility of the macromolecules can be significantly decreased. The dense cross-linking can transfer the polyisoprene viscoelastic state to the elastic state up to a high modulus material. Due to the carbon black surface being the source of the free radicals, a gradient of free radicals concentration from the maximum near the carbon black surface and gradually decaying with the distance from the carbon black surface is expected. The modeling of the free radical reaction showed that the closest polyisoprene layer has a maximal cross-linking density. Therefore, the closest layer is expected to be a solid with low mobility and a high modulus. The next layer has fewer cross-links and is softer than the closest layer. The macromolecular mobility of this layer is higher than the closest layer, but lower that the bulk layer of polyisoprene far from the particles. This fact supports the rubber models with the specific “hard glassy layer” and “sticky hard layer” near the carbon black particles. Such models explain the mechanics of the rubber materials, in particular, the Mullins effect and high strength of the rubber filled with the carbon black particles.

The significant changes can be observed at the loading of such cross-linked layers of polyisoprene. The free radicals can remain active in the polymer material for a long time. This effect is called “trapped radicals”, which is well known in electret materials. The mechanical deformation of the polymer consisting of the free radicals can change the conformation of the macromolecules and ignite the additional chemical reactions of the trapped radicals. One result of the polymer stretching in a presence of the radicals is the intensified scission of the polymer macromolecules. When such a polymer is released or compressed, the free radicals can cause additional cross-linking in the macromolecules.

A high filler concentration elastomer material is a multilevel complex system that requires a similarly complicated mathematical model. First, the model must be multilevel. The interactions between the polymer chains and between the polymer chain and the filler particle must be modeled. Second, the model requires an understanding the particle–particle interaction nature, where the geometry of the filler particles and their distribution in the rubber play significant role. The structure transformations in the rubber during the stretching also play a significant role in the rubber mechanics. One of the bright observations of such structural transformations during the stretching is the Mullins effect, when the viscous–elastic properties of the rubber appear. All of these factors are complicated, and could not be described in one general model now. A way in which some effects could be separately modeled and then be united as separate blocks in the general model would be more promising. In the present study, we consider a process of the structure formation near the carbon black particles. The structure particularity of the macromolecules near the filler particles influence the particle–particle interactions, structural transformations during the stretching, the Mullins effect, viscous–elastic properties of the rubber, and finally the failure of the rubber.

The formation of a highly cross-linked region of the polyisoprene macromolecules between the filler particles plays an important role in the specific mechanical properties of the rubber materials. The combination of such highly cross-linked regions of the polyisoprene with low cross-linked regions remain highly elastic, which makes the filled rubber a unique material. When rubber is deformed, the densely cross-linked polyisoprene macromolecules between the carbon black particles are ruptured, while the rarely cross-linked polyisoprene macromolecules between the agglomerates that are far from the particles are deformed, but remain unbroken. As a result, the total modulus of the rubber decreases, while a crack in the rubber does not appear, and any damage to the rubber is not observed. The rubber becomes softer after deformation. This effect is known as effect of Mullins [30,31], and is a reason for the high wear resistance of the rubber with the carbon black filler that is used in all car tires, including the ones used in Formula 1 race cars. 

The cross-linking of macromolecules between the carbon black particles are due to free radicals migrating from the carbon black particles, and are observed in polyisoprene macromolecules. However, the cross-linking mechanism of the hydrocarbon macromolecules can be observed for other elastic rubbers based on polybutadiene or ethylene–propylene (EPDM) rubbers.

The free radicals in carbon black are sensitive to the environment, and can be deactivated in the presence of some substances. Also, the free radicals in the carbon black particles decay with storage time. This mechanism explains some of the technological requirements for the rubber manufacturing, such as a limited storage time and environmental conditions for the carbon black filler, the optimal temperature of rubber vulcanizing, and optimal pressure [1,2].

## 6. Conclusions

This study has demonstrated that the carbon black particles contain a number of free radicals on the surface, which migrate to the polyisoprene macromolecules adsorbed on the surface and cause the cross-linking of the macromolecules on a distance up to 30–35 nm from the particle surface. This cross=linked polyisoprene layer is interpreted as the bounded “glassy” rubber layer, and is exploited in a number of theoretical models for the rubber mechanics.
